# Complete genome sequence of *Agrobacterium fabrum* ARqua1

**DOI:** 10.1128/MRA.00554-23

**Published:** 2023-10-09

**Authors:** Bo Lan, Qian Zhang, Kangquan Yin

**Affiliations:** 1 School of Grassland Science, Beijing Forestry University, Beijing, Beijing, China; 2 Chinese Academy of Agricultural Sciences, Lanzhou Institute of Husbandry and Pharmaceutical Sciences, Lanzhou, China; University of Southern California, Los Angeles, California, USA

**Keywords:** genomes, *Agrobacterium*, genome editing

## Abstract

We report the complete genome of *Agrobacterium fabrum* ARqua1 generated from Oxford Nanopore and Illumina sequencing. The genome of ARqua1 has a total length of 5,714,310 bp, comprising a circular chromosome, a linear chromosome, and two plasmids. In total, 5,446 genes were predicted, of which 5,288 were annotated.

## ANNOUNCEMENT

*Agrobacterium fabrum* is a well-known natural agent that induces hairy root diseases in plants and has been widely used in transgenic technology (1 – 3). Among these, *A. fabrum* ARqua1 has been used for genetic transformation in more than 60 plant species (4). The ARqua1 is derived from strain R1000, which has the C58 chromosomal background from *A. tumefaciens* A136 (5). Recently, a draft shotgun assembly of *A. fabrum* ARqua1 consisting of 19 contigs was reported (6). This report describes the complete genome assembly of *A. fabrum* ARqua1, which will facilitate genetic engineering of the “engineer” and generation of improved *Agrobacterium* strains for plant transformation and genome editing (7).

ARqua1 strain was obtained from Shanghai Weidi Biotechnology Co. Ltd. The strain was streaked onto Luria broth (LB) plates, and a single colony was selected for amplification with streptomycin (100 mg/L). The same genomic DNA extracted using the cetyltrimethylammonium bromide (CTAB) method (8) was used for both Oxford Nanopore and Illumina sequencing. For Oxford Nanopore sequencing, DNA was sheared using g-TUBE, and large DNA fragments (>20 Kb) were selected using BluePippin (Sage Science, Beverly, MA). Nanopore libraries were prepared using the SQK-LSK109 Genomic DNA Ligation Kit (Oxford Nanopore Technologies, UK) and sequenced on an FLO-PRO002 R9.4.1 flow cell. The basecalling and adapter trimming were performed using Guppy version 3.2.6. Read QC was performed using fastp v0.23.1 (9). Reads with quality scores less than 6.0 or shorter than 2,000 bp were filtered. Nanopore sequencing yielded 101,392 clean reads and an N50 of 26,601 bp. Illumina libraries were prepared using the TrueLib DNA Library Rapid Prep Kit and sequenced on an Illumina NovaSeq 6000 (Illumina, San Diego, CA, USA). Illumina sequencing yielded 12,333,606 paired-end reads (mean read length, 147 bp), which were trimmed using fastp version 0.23.1 (9), resulting in a total of 988,367,215 bp (~173-fold coverage). Nanopore sequencing data were used for *de novo* genome assembly using the Canu version 1.5 (10), and the post-assembly correction was conducted using Racon version 3.4.3 (11). Illumina sequencing data were used for further correction using Pilon version 1.22 (12). Circularization of chromosomal and plasmid contigs was conducted using Circlator version 1.5.5 (13) with the parameter “minimus2 --no_pre_merge.” Genome was annotated using Prodigal version 2.6.3 (14), RepeatMasker version 4.0.5 (15), Infernal version 1.1.3 (16), and tRNAscan-SE version 2.0 (17), respectively. Functional annotation of genes was conducted using eggNOG version 4.0, Pfam version 32.0, Swissprot access date 2019-07-31, and TrEMBL access date 2019-07-13 databases. Collinearity analysis was performed using the One Step MCScanX plugin in TBtools version 1.120 (18). Default parameters were used for all of the above software unless otherwise specified.

**Fig 1:**
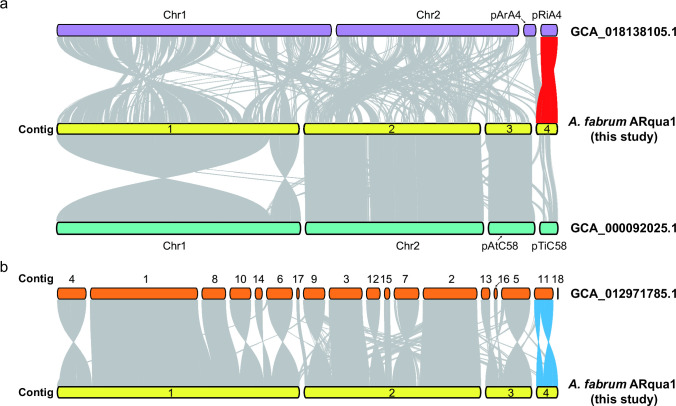
Genome synteny maps. (**a**) Synteny links between current *A. fabrum* ARqua1 genome and *A. rhizogenes* A4 genome (GCA_018138105.1, https://www.ncbi.nlm.nih.gov/datasets/genome/GCF_018138105.1/), and synteny links between current *A. fabrum* ARqua1 genome and *A. fabrum* C58 genome (GCA_000092025.1, https://www.ncbi.nlm.nih.gov/datasets/genome/GCF_000092025.1/). The red lines indicate the synteny links between the plasmid pRiA4 of *A. rhizogenes* A4 and contig 4 of the current *A. fabrum* ARqua1 genome. (**b**) Synteny links between the current *A. fabrum* ARqua1 genome and the first draft genome of *A. fabrum* ARqua1 (GCA_012971785.1, https://www.ncbi.nlm.nih.gov/datasets/genome/GCF_012971785.1/). The blue lines indicate the syntenic links between contig 11 of the draft genome of *A. fabrum* ARqua1 and contig 4 of the current *A. fabrum* ARqua1 genome.

The assembly resulted in four contigs: a circular chromosome, a linear chromosome, and two plasmids. The assembled genome had a GC content of 59.03%. Collinearity analysis showed that contig4 of the current ARqua1 genome was plasmid pRiA4b and confirmed that the chromosomal background of the ARqua1 strain was *A. fabrum* C58 (Fig. 1).

## Data Availability

The Bioproject number is PRJNA976066. The SRA accession numbers are SRR24759617 (Illumina) and SRR24759618 (ONT).
